# Mothers’ health care seeking behavior and associated factors for common childhood illnesses, Northwest Ethiopia: community based cross-sectional study

**DOI:** 10.1186/s12913-019-3897-4

**Published:** 2019-01-23

**Authors:** Muluye Molla Simieneh, Mezgebu Yitayal Mengistu, Abebaw Addis Gelagay, Mulugeta Tesfa Gebeyehu

**Affiliations:** 1 Department of Public Health, College of Health Sciences, Debre Markos University, P. O. Box 269, Debre Markos, Ethiopia; 2 Institute of Public Health, College of Medicine and Health Sciences, University of Gondar, Gondar, Ethiopia; 3 Institute of Public Health, College of Medicine and Health Sciences, University of Gondar, Gondar, Ethiopia; 4 Department of Public Health, College of Health Sciences, Debre Markos University, Debre Markos, Ethiopia

**Keywords:** Health care seeking behavior, Childhood illness, Mothers/care givers, Ethiopia

## Abstract

**Background:**

Substantial progress has been made in reducing child mortality over the last decades, however the magnitude of the problem is yet high globally Appropriate health care-seeking behavior of mothers/guardians for common childhood illnesses could prevent a significant number of child deaths and complications due to childhood illnesses, currently, there is few of studies in Ethiopia. Therefore, this study aimed to assess mothers/caregivers health care seeking behavior for their children in Northwest Ethiopia.

**Methods:**

Community based cross-sectional study of rural mothers living in Aneded district from February to March 2016. Data were collected using structured questionnaire by an interviewer. Bivariate and multivariable logistic regression analyses were performed to identify factors associated with health care seeking behavior. Variables having *P* value ≤0.2 in the bivariate analysis were considered for multivariable analysis. *P*-value less than 0.05 was used to declare that there was statistically significant association. Odds Ratio (OR) with 95% confidence interval (CI) was used to determine the strength and direction of association.

**Result:**

A total of 410 mothers participated in this study. Among 48.8% (95% CI: 44, 53.6%) had sought health care, only 27% sought health care within a day. Having awareness of childhood illness (AOR = 3.8, 95% CI: 2.18–6.72), perceived importance of early treatment (AOR = 3.5, 95% CI: 2.00–6.07) and child age <  24 months (AOR = 1.7, 95% CI: 1.08–2.68) and illness not being perceived as severe (AOR:= 0.17, 95% CI: 0.09–0.30) were all factors associated with mothers healthcare seeking behavior during their child illness.

**Conclusion:**

Overall health care seeking behavior level was low. Awareness, perceived illness severity, perceived early treatment and having young children were predictors of mothers’ health care seeking behavior. The Woreda health office administrators and health professionals should work to improve mothers’ awareness and perception towards childhood problems and the importance of early seeking appropriate health care using the existed structures (one-to-five women networking and health developmental army).

**Electronic supplementary material:**

The online version of this article (10.1186/s12913-019-3897-4) contains supplementary material, which is available to authorized users.

## Background

There has been substantial progress in reducing child mortality globally in the last decades. However, the magnitude of child mortality is high yet. Globally, around 5.6 million children under five died in 2016 [1, 2]. There is a huge gap in child mortality rates between high income and low income countries. In 2016, the under five mortality rate in low income and high income countries was 73.1 and 5.3 deaths per 1000 live births respectively. This represents about 14 times higher in low income countries [3]. In 2016, the under five mortality rate in Ethiopia was 67 deaths per 1000 live births [4].

Reducing child mortality is a worldwide health priority and one of global sustainable development goals. By 2030, it has been planned to end preventable deaths of newborn and children under 5 years of age and to reduce under five mortality to as low as 25/1000 live births [3]. Ethiopia is one country that has adopted this goal [5].

Self medication has public health importance mainly in sub-Saharan Africa [6]_._ Which contributes to delays in accessing appropriate heath care within the formal health sector which in-turn worsens child’s health problems [7].

In order to decrease severity of childhood illnesses and its subsequent death, improving access to skilled health professionals [8] and appropriate health care-seeking behavior of mothers [9] are critically important. Despite there is a substantial investment in health in Ethiopia, utilization of maternal and child care remain low [10]. In Ethiopia, only a small proportion of children with common childhood illnesses receive appropriate heath care. This problem is particularly pronounced in rural mothers [11, 12]. The 2016 EDHS report showed that only 30, 35, and 44% of children with symptoms of acute respiratory infection (ARI), fever, and diarrhea sought treatment respectively [4]. The health care seeking behavior of mothers or guardians for common childhood illnesses may not be the same across the regions and districts in Ethiopia. This may vary by the local contexts for example differences in socio-demographic and economic characteristics of the community affects their uptake of health care services. Assessing health care seeking behavior and identifying factors at a local (district) level are important to develop strategies and design appropriate interventions.

Therefore, this study aimed to assess the level of health care seeking behavior and associated factors among rural mothers/care givers during their child’s illness. Hence, the findings of this study will be used by health care planners and health professionals to take appropriate measures.

## Methods

### Study design and area

A community based cross-sectional study was conducted in Aneded district from February to March 2016. Aneded district located 283 km from Addis Ababa, the capital city of Ethiopia, and 282 km from Bahir Dar, the capital city of the Amhara National Regional State. The 2007 Ethiopian census reported the population to be 104,053 (50,991 males and 53,062 females) and the total number of children under-five at 12,351. It has 19 rural Kebeles and one urban kebele (the smallest administrative unit) and each Kebele is divided into Ketenas. There are 5 health centers, 20 health posts, 3 private clinics and 2 drug stores/pharmacies in the district. Health centers are public institutions that provide diagnostic and therapeutic services, staffed by mid-level health professionals (Diploma and Degree level health professionals). Ideally, five health posts within each health center. Health posts are staffed by health extension workers and low level health professionals. Staff working at health centers and health posts can assess and treat a sick children using integrated management of neonatal and childhood illnesses (IMNCI) protocols. The clinics in the district provide diagnostic and therapeutic services while the drug stores/pharmacies are established to sale drugs/medicines.

### Sample size determination and sampling procedures

The sample size was determined using two population proportion formula by considering different factors that affected mothers’ health care seeking behavior during child illness from previous study with Open Epi software version 2.3. To find adequate sample size, we took factor that gave largest sample size among the factors. Hence, the final sample size was calculated by considering (P_1_ = 69%) the proportion of media exposed mothers who sought health care and (P_2_ = 47.7%) the proportion of non-media exposed mothers who sought health care for their children illnesses [13], 5% level significance, 80% power, design effect of 2 and 10% non response rate. Therefore, the total calculated sample size was 410 mothers.

Mothers or caregivers who were living in rural district and who had a child or children under 5 years of age with history of any common childhood illness like diarrhea, fever, and/or ARI three months preceding the survey were included. Then, to select study participants, multistage cluster sampling technique was used. Among the 19 rural Kebeles in the district, five Kebeles (25% of the study area) namely Gudalem, Amberzura, Daget, Yewobie, and Nefasam were selected first using simple random sampling technique (lottery method) and then a minimum of 50 % of Ketenas from the selected Kebeles were selected using a simple random sampling method. The sample size was proportionally allocated to each kebele by considering the total population in each kebele. Since cluster sampling technique was used, households were visited to assess the presence of under five children who were sick within the past three months. Each households were visited until the sample size was reached. Mothers or guardians who had had sick child in the past three months were requested to participate in this study after providing adequate information and obtaining informed verbal consent. Data were collected using interviewer administered pre-tested structured questionnaire. The questionnaire draws from Andersen’s Behavioral Model [14] and a review of relevant published literature. In Andersen’s Behavioral Model access to and use of health services is considered a function of three characteristics: 1) Predisposing factors: the socio-cultural characteristics of individuals that exist prior to their illness, 2) Enabling factors: the logistical aspects of obtaining care for personal /family, 3) Need factors: the most immediate cause of health service use, perceived need and refers to how people view their general health, functional state and judgment to seek professional help (Additional file 1).

Five data collectors and two supervisors (nurses) were involved in the data collection. To assure data quality, a one day training was given to the data collectors and supervisors on the study objectives and data collection techniques. The overall data collection activity was supervised by study investigators.

### Operational definitions/measurements

The outcome variable was mothers’/caregivers’ health care seeking behaviors during childhood illness. The response was dichotomized as “yes” when women had appropriate health care seeking behavior or “no” when they did not have appropriate health care seeking behavior.

Appropriate health care seeking behavior was defined as situations when women visited any health facility/institution (governmental or private or both) during common childhood illnesses. Conversely, inappropriate refers to situations when a women did not visit any of formal health sectors.

Common childhood illnesses: include diarrhea, acute respiratory infection (ARI) and fever.

Awareness of childhood illness: refers to when a mother/care giver recognizes one or more symptom questions.

Mothers’ awareness on sign of severity of illnesses: refers to when a mother/care giver mentioned one or more of the signs of a sever childhood illnesses.

Perceived illness severity: refers to when a women/care giver thought that her sick child was severely ill.

In this study, the authors dichotomized the wealth of the participants that the term ‘rich’ was used to describe those who were in the fourth or fifth quintile where as the term ‘poor’ was used to explain those who were the first three quintiles.

The informal sector refers to institutions that are not legally able to diagnosis and treat childhood illnesses and includes local/traditional healers and Holy water. The formal health sector (health care system) are public health institutions and licensed private clinics able to diagnosis and treat childhood illnesses.

### Data processing and analysis

During data collection, supervisors and investigators manually checked the questionnaires daily for completeness. The collected data were entered into Epi- Data version 3.1 and exported to SPSS version 20 for data cleaning and analysis. Descriptive statistics was computed to summarize the descriptive results and presented in texts, graphs and charts. Principal component analysis (PCA) was employed to measure the level of wealth of the household. Multivariable logistic regression modeling used to identify factors associated with mother/caregiver health care seeking behavior. David W. Hosmer and Stanley Lemeshow in their second edition book entitled “Applied Logistic Regression” recommended to use a *P*-value of less than 0.25 as a screening criteria for variable selection for the multivariable analysis [15]. There are also other published articles in BMC journals which used a *p*-value of 0.2 as a cut-off point to select variables for the multivariable analysis [16–18]. Therefore, in this study, variables having *P*-value ≤0.2 in the bivariate analysis were considered for multivariable analysis. Odds Ratio with 95% confidence interval (CI) was used to determine the presence of an association.

### Ethical considerations

Ethical approval was obtained from the Ethical Review Board (IRB) of University of Gondar, Institute of Public Health. Support letters were obtained from Amhara Regional State Health Bureau, East Gojjam Zonal Health Department and Aneded District Health Office. Verbal consent was obtained from each study participant.

## Results

### Socio-demographic and economic characteristics of respondents

A total of 410 mothers/caregivers participated in this study. More than half (57.8%) of them were 25–34 years old and the mean age of mothers was 30.5 (SD+ 6.3) years. The majority (90%) of respondents were married. All of the mothers/care givers were Amhara by Ethnicity and Orthodox Christian in religion. Regarding educational status, 86.8% of the mothers and 39.2% of their husbands were unable to read and write. Almost all (90%) of respondents were house wives. The average family size was 5 children with range of 2–11 (Table 1).

**Table 1:** Socio-demographic and economic characteristics of mothers or caregivers, Aneded District, North West Ethiopia, 2016

Variable (*n* = 410)		Frequency	Percent (%)
Age of the mothers	18–24 years	58	14.1
	25–34	237	57.8
	35–49	111	27.1
	50+	4	1.0
Marital status	Married	369	90.0
	Single	28	6.8
	Divorced	12	2.9
	Widowed	1	0.2
Family size	< 5	162	40
	5–7	215	52.4
	8+	33	8.1
Mothers` occupation	House wife	369	90.0
	Farmer	29	7.1
	Merchant	10	2.4
	Daily laborer	2	0.5
Education of mothers	Unable to read and write	356	86.8
	Able to read and write	54	13.2
Husbands’ education (*n* = 369)	Unable to read and write	154	39.2
	Able to read and write	215	60.8

### Mothers’/caregivers’ health care seeking behavior during child illness

**Fig. 1:**
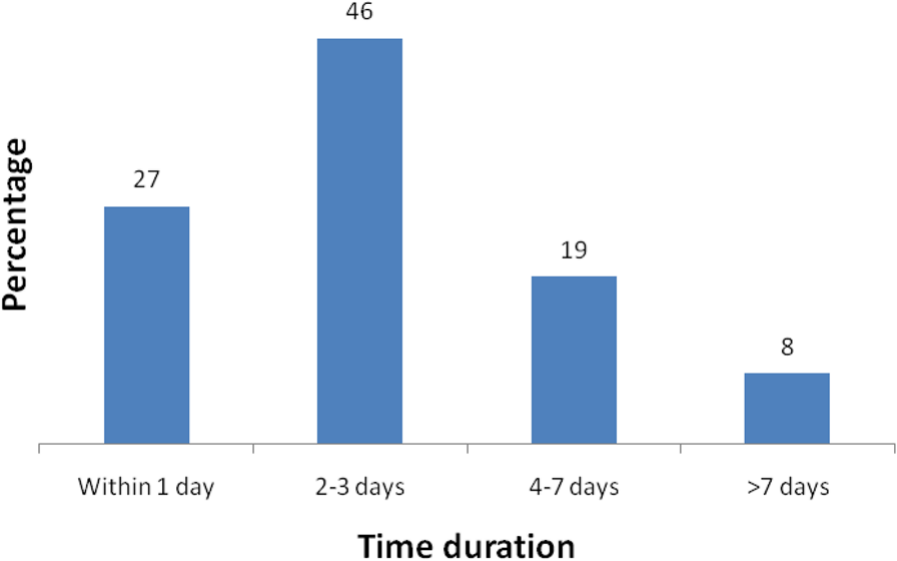
Time to care seeking after onset of their children illnesses, Aneded District, North West Ethiopia, 2016

**Fig. 2:**
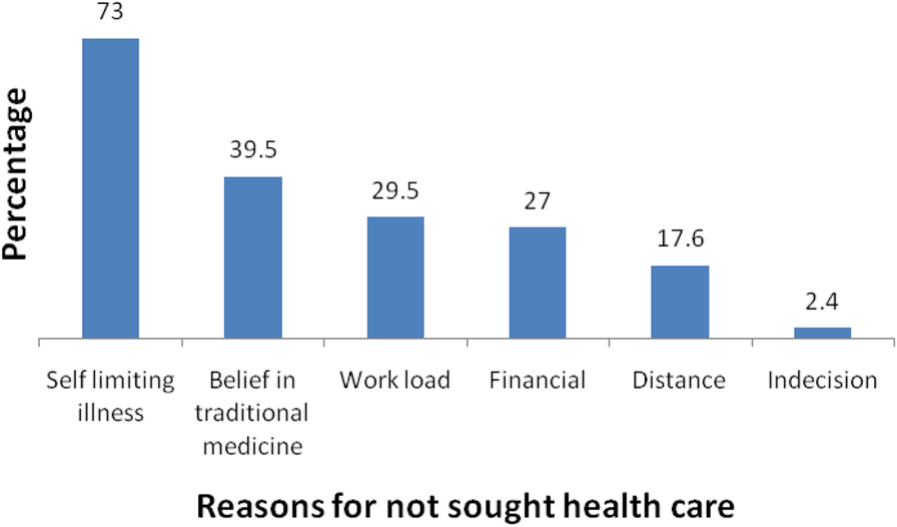
Reasons for not seeking healthcare for child’s illness, Aneded District, Northwest Ethiopia, 2016

Among the study participants, less than half, 48.8%, (95% CI: 43, 53%), sought health care at health institutions during their children illnesses. Health posts and health centers were the most common sources of health care services as 84% of the mothers who sought health care visited these institutions. Among them, few mothers, 54(27%), sought health care within a day of observing signs of child illness (Fig. 1). Mothers’ main reason for not seeking appropriate heath care when their children got sick was their perception that the disease would resolve by itself (73%) (Fig. 2). Three-fourth (74.2%) of the mothers/caregivers perceived that children need health care early regardless of disease type. Around half of the respondents reported that their child/children had fever and cough or difficulty of breathing. Only 18.5% of respondents decided on their own to seek medical care for their child’s illness. Slightly more than three-fourth of the participants perceived that a child is severely ill when the child was unable to eat or breast feed. A significant proportion of the respondents misperceived the cause of childhood illness. For example, 42.2% perceived that it is due to curse (Table 2).

**Table 2:** Mothers health care seeking behavior in Aneded District, North West Ethiopia, 2016

Variables	Frequency	%
Health care seeking behavior		
Health facility	200	48.8
Not seek at all	99	24.1
Buying drugs by themselves	46	11.2
Traditional healers	28	6.8
Homemade treatment	22	5.4
Holy water	15	3.7
Perceived need for seeking early health care		
Children need health care early regardless of disease type	304	74.2
Depends on the disease types	87	21.2
No need of seeking health care early at all	19	4.6
Reported common child illness in the past three months^a^		
Fever	213	52
Cough or difficulty of breathing	203	49.5
Diarrhea	185	45.1
Decision to seek heath care at health institution during child illness		
Mother	72	18.5
Father	57	14
Both father and mother	277	67.5
Awareness of signs indicating severe illness^a^		
When the child is unable to feed or suck breast	317	77.3
When a child shows behavioral change	256	62.4
When the disease stayed for days	118	33.7
Perceived cause of child illness^a^		
Curse from God	173	42.2
Micro-organisms	190	46.3
Food contamination	116	28.3
Evil eye	73	17.8

Remark: ^a^Indicates the sum in the above table could be greater than 100%, since it is a multiple response

### Factors associated with mothers’/caregivers’ health care seeking behavior for their children illnesses

In the bivariate analysis, child age, sex, mothers/caregivers awareness of childhood illnesses, perceived illness severity, wealth quintiles, perception on early treatment, and distance from heath institution were variables associated with seeking formal care. However, sex of child, wealth quintiles, and distance from heath institution had no statistically significant association in the multivariable association.

This study showed that mothers/caregivers who had children aged < 24 months were 1.7 times (AOR = 1.7; 95% CI: 1.08–2.68) more likely to seek health care compared to those who had children aged > 24 months of age. Mothers who were aware of common childhood illnesses were 3.8 times (AOR =3.8; 95% CI: 2.18–6.72) more likely to seek appropriate health care. Mothers who perceived that their child had a severe illness were 83% less likely (AOR = 0.17; 95% CI; 0.09–0.30) to seek appropriate health care than mothers/caregivers who perceived their child had a severe illness. Mothers reporting that children should be treated early during their illness (regardless of the disease type) were 3.5 times (AOR = 3.5; 95% CI 2.00–6.07) more likely to seek health care than their counterparts (Table 3).

**Table 3:** Bivariate and multivariable analysis for health care seeking behaviour for common childhood illnesses among mothers/care givers at Aneded District, North West Ethiopia, 2016

Variables		Seek health care		COR(95% CI)	AOR(95% CI)
		Yes	No		
Child age	< 24 months	108	91	1.54 (1.04–2.27)	1.70 (1.08–2.68)*
	> 24 months	92	119	1	1
Sex of child	Male	108	98	1.34 (0.91–1.98)	
	Female	92	112	1	
Awareness on Childhood illness	yes	173	132	3.79 (2.31–6.20)	3.83 (2.18–6.72)**
	No	27	78	1	1
Perceived illness severity	Not sever	125	185	0.2 3 (0.14–0.37)	0.17 (0.09–0.30)**
	Sever	75	25	1	1
Dichotomized wealth^***^	Poor	109	137	0.64 (0.43–0.95)	
	Rich	91	73	1	
Mothers’ perception on early treatment regardless of disease type	Yes	170	134	3.21 (1.99–5.19)	3.48 (2.00–6.07)**
	No	30	76	1	1
Distance from heath institution	<= 5 kms	135	124	1.44 (0.96–2.16)	
Distance from heath institution	<= 5 kms	135	124	1.44 (0.96–2.16)	
	>5kms	65	86	1	

*Significant at *P* < 0.05 **Significant at *P* < 0.001 ***Poor = quintile 1–3, rich = quintile 4–5

## Discussion

Improving health care-seeking behavior of mothers for childhood illnesses can help reduce child mortality and morbidity [9]. This study revealed that only nearly half (48.8%) of participants sought health care for their children during common childhood illness. This is consistent with a prior study conducted in Amhara region (49.6%), but lower than a survey conducted in Nairobi (65%) [19]. The possible reason for the observed differences might be due to socio- demographic differences. Our finding was also lower than the finding of a survey conducted in Bahir Dar (82.7%) [20]. This might result from differences in study settings. This study was conducted in mothers living in a rural environment while the previous work was conducted in urban settings. This could introduce variations in health care seeking behavior during their child’s illness. Among mothers who sought health care at health institutions, few (27%) sought health care within 24-h after they recognized their child’s illness. This is consistent with a study conducted in rural communities of Osun State, south-western Nigeria. The possible explanation for this might have been due to mothers’ perception that the disease was self limiting.

Mothers/caregivers who had children aged < 24 months were more likely to seek appropriate health care compared to those with older children. This is similar to studies in Bangladesh [21], rural Nigeria [22], rural Tanzania [23], Sub-Saharan Africa [24], and Ethiopia [25]. This might have been due to mothers’ understanding that children illnesses were more severe in younger compared to older children (> 2 years).

Mothers who perceived children should be treated early during their child illness were more likely to seek appropriate health care. This finding is supported by a study in Kenya [26]. Mothers/caregivers who were aware of common childhood illnesses were more likely to seek health care than those mothers who were not. This is similar to results of a systematic review conducted in developing countries [27]. Recognizing danger signs of childhood illnesses is an important factor that motivates mothers/care givers to take medical help. This study showed that mothers who did not perceive severe illness were less likely to seek health care than those mothers who perceived the illness as severe, in line with a systematic review in developing countries [28] as well as studies done in western Nepal [29], rural Nigeria [22], Kenya [26, 30], and Ethiopia [12, 25]. In this study, distance from health institutions was not significantly associated with seeking appropriate health care. This is in contrast to studies in rural Ensaro District, North Shoa Zone [31] in south-west Ethiopia [32]. This might be due to all of the study participants in this study were found on average within a 5 km distance from the nearest health institution. Similarly, in this study, income was not significantly associated with mother/caregiver health care seeking behavior. This is inconsistence with studies conducted in a Nigerian Teaching Hospital [33], and a Tribal Community of Gujarat, India [13]. This might be due to the fact that, in the present study, income was not barrier to seek health care as services are provided free of charge for children under the age of five years. Since the data were collected by interviewers, the data might be affected by social desirability bias. This is a limitation of this study. However, the authors tried to minimize this by training the data collectors to provide adequate information e and emphasize importance of honesty in the survey responses.

## Conclusions

The proportion of mothers who sought appropriate health care during their child’s illness was low. Awareness of common childhood illnesses, perceived illness severity, perception on early treatment and child age <  24 months were positively associated with mothers’ or care givers’ health care seeking behavior. These findings suggest a need for interventions aimed at improving mother/caregiver awareness and perception of common childhood illness. Further, the findings implicate the Woreda (district) health office, healthcare professionals, health extension workers, health development army, and local “one-to-five” community groups as key stakeholders. A variety of community-based platforms could be used to accomplish this including gatherings, home visits, and through existing community services.

## Additional file

Additional file 1: Questionnaire- a questionnaire we used to collect the data. (DOCX 33 kb)

## Abbreviations

AOR: Adjusted Odds Ratio
ARI: Acute Respiratory Infection
CI: Confidence Interval
EDHS: Ethiopian demographic and health survey
HSB: Health Care Seeking Behavior
Kms: Kilometers
OR: Odds Ratio
SPSS: Statistical Package for Social Science

## References

[1] WHO (2016). Global strategy for Women's, Children's and Adolescents' health (2016–2030).

[2] UNICEF (2017). Levels and trends in child mortality 2017.

[3] WHO, Under five Mortality 2018.

[4] Central Statistical Agency (CSA)[Ethiopia] and ICF., Ethiopia Demographic and Health Survey 2016*.* Addis Ababa, Ethiopia, and Rockville, Maryland, USA: CSA and ICF*.* July 2017,.

[5] UNICEF:, Committing to Child Survival Progress Report 2012. United Nations Plaza, New York, USA: UNICEF;, in United Nations Plaza, New York*,*. 2012.

[6] Diaz T (2013). Healthcare seeking for diarrhoea, malaria and pneumonia among children in four poor rural districts in Sierra Leone in the context of free health care: results of a cross-sectional survey. BMC Public Health.

[7] Schoeps A (2015). Health insurance and child mortality in rural Burkina Faso. Glob Health Action.

[8] World Health Organization, Causes of child Mortality 2016.

[9] Adedire EB, Asekun-Olarinmoye EO, Fawole O. Maternal perception and care-seeking patterns for childhood febrile illnesses in rural communities of Osun state, South-Western Nigeria. Sci J Public Health. 2015;2(6):636–43.

[10] Mebratie A, et al. Self-reported health care seeking behavior in rural Ethiopia: evidence from clinical vignettes. Inst Social Stud. 2013; ISSN 0921-0210.

[11] Deressa W, Ali A, Berhane Y (2007). Maternal responses to childhood febrile illnesses in an area of seasonal malaria transmission in rural Ethiopia. Acta Trop.

[12] Assefa T, et al. Mothers’ Health Care Seeking Behavior For Childhood Illnesses In Derra District, Northshoa Zone, Oromia Regional State, ETHIOPIA. Ethiop J Health Sci. 2008;18(3):87–94.

[13] Chandwani IH, Pandor J (2015). Healthcare-seeking behaviors of mothers regarding their children in a tribal Community of Gujarat. Electron physician.

[14] Andersen RM, Newman JF (1995). Andersen and Newman framework of health services utilization. Health Serv Res.

[15] Hosmer DW, Lemeshow S (2000). Applied Logistic Regression.

[16] Gebremedhin AY (2018). Family planning use and its associated factors among women in the extended postpartum period in Addis Ababa, Ethiopia. Contracept Reprod Med.

[17] Alemayehu GA (2018). Prevalence and determinants of contraceptive utilization among married women at Dabat health and demographic surveillance system site*,* northwest Ethiopia. BMC Women's Health.

[18] Gelagay A. A, Koye D.N., and Yeshita H. Y, Demand for long acting contraceptive methods among married HIV positive women attending care at public health facilities at Bahir Dar City*,* Northwest Ethiopia Reprod. Health 2015. 12: p. 76. 10.1186/s12978-015-0073-0 PMC4551468 26311141

[19] Robert F. Breiman, et al., Healthcare-use for Major Infectious Disease Syndromes in an Informal Settlement in Nairobi, Kenya*.* International Centre For Diarrhoeal Disease Research, Bangladesh, 2011 29(2): p. 123–133. 10.3329/jhpn.v29i2.7854 PMC3126984 21608421

[20] Awoke W (2013). Prevalence of childhood illness and mothers’/caregivers’ care seeking behavior in Bahir Dar, Ethiopia: a descriptive community based cross sectional study. Open J Preventive Med.

[21] Sarker AR (2016). Prevalence and health care–seeking behavior for childhood diarrheal disease in Bangladesh. Global Pediatr Health.

[22] Abdulraheem IS, Parakoyi DB (2009). Factors affecting mothers’ healthcare-seeking behaviour for childhood illnesses in a rural Nigerian setting. Early Child Dev Care.

[23] Kanté AM (2015). Childhood Illness Prevalence and Health Seeking Behavior Patterns in Rural Tanzania. BMC Public Health.

[24] Noordam AC (2015). Care seeking behaviour for children with suspected pneumonia in countries in sub-Saharan Africa with high pneumonia mortality. POLS ONE.

[25] Gelaw YA, Biks GA, Alene KA (2014). Effect of residence on mothers’ health careSeeking behavior for common childhood illness in Northwest Ethiopia: a community based. BMC Res Notes.

[26] Burton DC (2011). Healthcare-seeking behaviour for CommonInfectious disease-related illnesses in rural Kenya: ACommunity-based house-to-house survey. J Health Popul Nutr.

[27] Geldsetzer P, et al. The recognition of and care seeking behaviour for childhood illness in developing countries: a systematic review. PLoS One. 2014;9(4):e93427. 10.1371/journal.pone.0093427 PMC3981715 24718483

[28] Geldsetzer P, et al. The Recommendation of and Care Seeking Behavior for Childhood Illness in Developing Countries: A Systematic Review. PLoS ONE, 2014.;9(4):e93427 10.1371/journal.pone.0093427 PMC3981715 24718483

[29] Sreeramareddy CT (2006). Care seeking behaviour for childhood illness- a questionnaire survey in western Nepal.

[30] Mbagaya GM (2005). Mother’s health seeking behaviour during child illness in a rural western Kenya community. Afr Health Sci.

[31] Sisay S, Endalew G, Hadgu G (2015). Assessment of mothers/care givers health care seeking behavior forChildhood illness in rural Ensaro District, north Shoa Zone, Amhara region, Ethiopia 2014. GJLSBR.

[32] Deribew A, Getahun A, Deribe K (2010). Determinants of delay in malaria treatment-seeking behaviour for under-five children in south-West Ethiopia: a case control study. BMC.

[33] Ajibade BL, et al. Determinants of Mothers Health Seeking Behaviour for their Children in a Nigerian Teaching Hospital. J Nursing and Health Sci (JNHS). 2013;1(6):9–16.

