# Supplementary material for: Mothers’ health care seeking behavior and associated factors for common childhood illnesses, Northwest Ethiopia: community based cross-sectional study
Source: BMC Health Serv Res. 2019 Jan 23;19:59. doi: 10.1186/s12913-019-3897-4 (PMC6343298; doi:10.1186/s12913-019-3897-4)
Supplement: Supplementary file 1 — Questionnaire- a questionnaire we used to collect the data. (DOCX 33 kb) [file 12913_2019_3897_MOESM1_ESM.docx]

**Information sheet and consent form.**

Assessment /study group: Mothers` or care givers`

Information sheet and consent forms prepared for mothers or care givers health care seeking behavior and associated factors during under five childhood illness in Aneded District .

**Introduction:** This information sheet is prepared by the research team whose main aim to study mother or care givers health care seeking behavior and associated factors during under five childhood illness in Aneded district.

**The purpose:** The purpose of this study is to assess mothers or care givers health care seeking behavior and associated factors of during under five childhood illness in Aneded district. This is an interview that target rural mothers who have under five children in Aneded district. Your response are very important, and will be helpful in planning for the necessary support toward improving appropriate health care seeking practices of mothers’ or care givers’ during under five child hood illness

**Procedure:** in order to assess mothers or care givers on health care seeking behavior and associated factors during under five childhood illness in Aneded district, we invite you to take part our project ,if you are willing to participate in our project, you need to understand and sign in consent form. Then, you will be asked to give your response for data collector for this questionnaire based study, participants are mothers’ or care givers’ who have under five children and living in the Aneded district . All responses given by the participants, and the results obtained will be kept anonymous and confidential using coding system whereby no one will have access to your responses.

**Risk and/or Discomfort:** By participating in this research there is no risk. But, you may feel that it has some discomfort specially on wasting few minutes but this may not be too much comparing its potential benefits that will contribute to improve appropriate health care seeking practices~~.~~

**Benefits:** If you participate in this research project, you may not get direct benefit but your participation is likely to help us in assess health care seeking behavior and associated factors for common childhood illness among mothers’ or care givers’ in Aneded district. It will also give an insight about the mother Health Care Seeking Behavior based on the findings of the study for improving the health status of the community

**Incentives:** You will not be provided any incentives to take part in this project.

**Confidentiality and Anonymity:** The information that we will collect from this research project will be kept confidential. Information about you that will be collected from the study will be stored in a file, which will not have your name on it, but a code number assigned to it. The number belongs to which name will be kept under lock and key, and it will not be revealed to anyone except the principal investigator.

**Right to Refuse or Withdraw**: You have the full right to refuse from participating in this research (you can choose not to respond for some or all of the questions) if you do not wish to participate; and this will not affect your health services you get from any health facilities. You have also the full right to withdraw from this study at any time when you wish without losing any of your rights as a beneficiary of health services.

**Consent for**

I have informed about all information and procedures that are part of this research study and I am interested to be a participant in this study. I understand that the research will be imposes no risk on my life and therefore no compensation would be provided. I hereby agree to participate in this research study and give my voluntary consent. I hereby also give rights to the researcher for collecting the data that are required for this study.

Agreed ________ Disagreed ________

Name of the interviewer: _________ Sign. _____ Date of interview ______

Name of the supervisor: ________ Sign. _______ Date ________

**English version questionnaires**

Before you go to your interview ask these questions.

- Is there under five child in this household? 1. Yes 2. No

If **``No``, stop** your interview and go to the next household.

- If ``**Yes``,** does the child have sickness in the past three months? 1. Yes 2. No,

If **``No``, stop** your interview and go to the next household.

If ``**Yes``,** **continue** your interview.

**Part I: Socio-Demographic and Economic Data**

**1.1. Socio-Demographic Data**

| **S.No** | **Variables** | **Alternatives** |
| --- | --- | --- |
| 101 | Age of mother or care giver | ______ years |
| 102 | Age of the child | ______ months |
| 103 | Sex of the child | 1. Male 2. Female |
| 104 | Marital status | 1. Single 2. Married 3. Divorced 4. Window |
| 105 | Ethnicity mother or care giver | 1. Amhara 2. Awi 3. Tigray 4. Oromo |
| 106 | Religion of mother or care giver | 1. Orthodox **Christian** 2. Muslim 3. Protestant 4. Catholic |
| 107 | Educational status of mother or care giver | 1. Unable to read and write 2. Read and write 3. Grade 1-4 4. Grade 5-8 5. Grade 9-10 6. Grade 11-12 7. Certificate and above |
| 108 | Educational status of partner | 1. Unable to read and write 2. Read and write 3. Grade 1-4 4. Grade 5-8 5. Grade 9-10 6. Grade 11-12 7. Certificate and above |
| 109 | Occupational status of mothers’ or care giver’ | 1. Farmer 2. House wife’s 3. Merchant 4. Labor worker 5. Student 6. Other (specify….............) |
| 110 | Occupational status of partner | 1. Farmer 2. Merchant 3. Labor worker 4. Student 5. Other (specify….............) |
| 111 | Number of family members | _______ in numbers |
| 112 | Number of children less than five years in the family | _______ in numbers |

**1.2. Wealth quintile**

| 113 | Type of the house | 1. Corrugated iron sheet 2. Thatch 3. Other specify --------------- |  |
| --- | --- | --- | --- |
| 114 | Availability of radio | 1. Yes 2. No |  |
| 115 | Availability of mobile telephone | 1. Yes 2. No |  |
| 116 | Availability of chair | 1. Yes 2. No |  |
| 117 | Availability of table | 1. Yes 2. No |  |
| 118 | Availability of bed with cotton/ sponge/spring matters | 1. Yes 2. No |  |
| 120.1 | Source of light is electricity | 1. Yes 2. No |  |
| 120.2 | Source of light is kerosene lamp/pressure lamp | 1. Yes 2. No |  |
| 120.3 | Source of light is kerosene lamp local (``kuraz`` | 1. Yes 2. No |  |
| 120.4 | Source of light is solar | 1. Yes 2. No |  |
| 120.5 | Source of light is battery | 1. Yes 2. No |  |
| 120.6 | Source of light is battery | Other………………. |  |
| 121.1 | Source of water is pipe water | 1. Yes 2. No |  |
| 121.2 | Source of water is well water | 1. Yes 2. No |  |
| 121.3 | Source of water is spring water | 1. Yes 2. No |  |
| 121.4 | Source of water is river water | 1. Yes 2. No |  |
| 121 | Does the household own any agricultural land? | 1. Yes 2. No |  |
| 122 | How many (local units) of agricultural land do you own? | 1. Private -------------------- (local unit) 2. Rent --------------------- (local units) |  |
|  | **Annual farm product per quintal** |  |  |
| 123 | Teff (you may not to all of the following but you can modify the list based on the availability of products in the study area) | ----------- quintal |  |
| 124 | Barley | ----------- quintal |  |
| 125 | Maize | ----------- quintal |  |
| 126 | cheek pea | ----------- quintal |  |
| 127 | Platypus | ----------- quintal |  |
| 128 | Wheat | ----------- quintal |  |
| 129 | Potato | ----------- quintal |  |
| 130 | Pea /bean | ----------- quintal |  |
| 131 | ``Nug`` /``Telba`` | ----------- quintal |  |
|  | **Availability of cattle’s** |  |  |
|  | How money of the following animal does the household own? | *You can change later to Yes/No or in money...* |  |
| 132 | Ox | ------------- in number |  |
| 133 | Cow |  |  |
| 134 | Calf |  |  |
| 135 | Horse | ------------- in number |  |
| 136 | Mules |  |  |
| 137 | Donkey | ------------- in number |  |
| 138 | Sheep | ------------- in number |  |
| 139 | Goat | ------------- in number |  |
| 140 | Chickens | ------------- in number |  |
| 141 | Beehives |  |  |

**Part II: - Health seeking behaviors of mothers or cares givers for common childhood illness.**

| **S.No** | **Variables** | **Alternatives** |  |
| --- | --- | --- | --- |
| 201 | How many times your child gets sickness in the past three months. | ----------------- times |  |
| 202 | What kind of symptom(s) you have observed when your child gets sick in the past three month? | 1. Cough 2. Difficulty of breathing 3. Fever 4. Diarrhea 5. If 6. others (specify)……………… | You can give more than one answer |
| 203 | What was your first choice when your child had got illness? | 1. Take to public health facilities 2. Take to private health facilities 3. Take to traditional treatment 4. Self-treatment at home 5. Treat the child with drugs brought from Pharmacies 6. Treat the child with drugs brought from drug sellers without prescriptions 7. Treat with holy water 8. Did nothing |  |
| 206 | If you took to HI, to which health institution you took your child first? | 1. Hospital 2. Health center 3. Health post |  |
| 207 | when did you took your child to HI while he/she got illness | 1. Within a day when symptom/s appeared 2. 2-3 days after symptom/s appeared 3. 4-7 days after symptom/s appeared 4. 7 days after symptom/s appeared |  |
| 208 | Reasons for delay/not go to health Institution with in a day | 1. Lack of money 2. Believe that the disease will be improved by itself 3. Believe that the disease will not be improved by treatment 4. Believe that the disease will be improve by traditional treatment 5. Long distance from HI 6. Lack of transport access 7. Lack of transport cost 8. Workload 9. Long waiting time in HI 10. Lack of self decision |  |
| 209 | What was the outcome of the child? | 1. Improved 2. Not improved 3. Dead |  |
| 210 | How long it takes from your home to the nearby health institution? | 1. Less than half an hour 2. Half an hour -1hr 3. 1:30-2hrs 4. 2hrs and above |  |
| 211 | Who decided to take the child for medical treatment? | 1. Mothers 2. Fathers 3. Both mothers and fathers 4. Grand parents |  |

**Part III: Mothers or cares givers awareness’ and perception on common childhood illness.**

| **S.No** | **Variables** | **Alternatives** |
| --- | --- | --- |
| 301 | According to your perception, how did you rate/see the degree of your child illness? | 1. Mild 2. Moderate 3. Severe |
| 302 | How did you identify the severity of illness on your child? | 1. My child refused to suck breast or eat foods 2. The illness continue for long time 3. When child behaviour change 4. Others (specify….) |
| 303 | Do you believe that children should go to health institutions immediately when they get sick? | 1. Yes 2. No |
| 304 | Do you believe that a child with diarrhea should go to health institution with in that day? | 1. Yes 2. No |
| 305 | Do you believe that a child with cough or difficulty of breathing should go to health institution with in that day? | 1. Yes 2. No |
| 306 | Do you believe that a child with fever should go to health institution with in that day? | 1. Yes 2. No |
| 307 | What do you think for the cause of the recent illness for your child? | 1. Curse from God 2. Micro-organisms 3. Evil eyes 4. Contaminated water and food 5. Others……………………. |
| 308 | Did you learn / know about signs and symptoms severity of the common childhood illness? | 1. Yes 2. No 3. It depends on the type of the disease |
| 309 | If yes, for question no 308 what are they | 1 -------------- 3-------------------  2 ----------------- 4------------------- |
| 310 | If yes, for question no 308, what was your source of information? | 1. Health care providers 2. Radio 3. Television 4. Family 5. Neighbor 6. Others |

Question End And Thank You For Your Participation!!

**QUESTION End AND THANK YOU for your participation!!**

# 
